# Arrhythmias in COVID-19/SARS-CoV-2 Pneumonia Infection: Prevalence and Implication for Outcomes

**DOI:** 10.3390/jcm11051463

**Published:** 2022-03-07

**Authors:** Andrea Denegri, Matteo Sola, Marianna Morelli, Francesco Farioli, Tosetti Alberto, Matteo D’Arienzo, Fulvio Savorani, Giuseppe Stefano Pezzuto, Giuseppe Boriani, Lukasz Szarpak, Giulia Magnani

**Affiliations:** 1 Cardiology Division, Parma University Hospital, 43125 Parma, Italy; gmagnani@ao.pr.it; 2 Faculty of Medicine & Surgery, University of Modena and Reggio Emilia, Via Università 4, 41121 Modena, Italy; sola.matte@gmail.com (M.S.); 212847@studenti.unimore.it (F.F.); 3 Emergency Department, Azienda Ospedaliero-Universitaria di Modena, Largo del Pozzo, 71, 41125 Modena, Italy; morelli.marianna@aou.mo.it (M.M.); darienzo.matteo@aou.mo.it (M.D.); savorani.fulvio@aou.mo.it (F.S.); pezzuto.giuseppe@aou.mo.it (G.S.P.); 4 Cardiology Division, Department of Biomedical, Metabolic and Neural Sciences, University of Modena and Reggio Emilia, Policlinic of Modena, 41125 Modena, Italy; albertotosetti1@gmail.com (T.A.); giuseppe.boriani@unimore.it (G.B.); 5 Henry JN Taub Department of Emergency Medicine, Baylor College of Medicine, Houston, TX 77030, USA; lukasz.szarpak@gmail.com; 6 Institute of Outcomes Research, Maria Sklodowska-Curie Medical Academy, 03-411 Warsaw, Poland

**Keywords:** arrhythmia, cardiovascular complication, SARS-CoV-2, COVID-19, mortality

## Abstract

Arrhythmias (ARs) are potential cardiovascular complication of COVID-19 but may also have a prognostic role. The aim of this study was to explore the prevalence and impact of cardiac ARs in hospitalized COVID-19 patients. All-comer patients admitted to the emergency department of Modena University Hospital from 16 March to 31 December 2020 and diagnosed with COVID-19 pneumonia infection were included in the study. The primary endpoint was 30-day mortality. Out of 902 patients, 637 (70.6%) presented a baseline 12-lead ECG registration; of these, 122 (19.2%) were diagnosed with ARs. Atrial fibrillation (AF, 40.2%) was the most frequent AR detected. The primary endpoint (30-day mortality) occurred in 33.6% (*p* < 0.001). AR-patients presented an almost 3-fold risk of mortality compared to non-AR-patients at 30d (Adj. OR = 2.8, 95%CI: 1.8–4.3, *p* < 0.001). After adjustment for significant baseline characteristics selected by a stepwise backward selection, AR-patients remained at increased risk of mortality (Adj. HR = 2.0, 95%CI: 1.9–2.3, *p* < 0.001). Sub-group analysis revealed that among ARs patients, those with AF at admission presented the highest risk of 30-day mortality (Adj. HR = 3.1, 95%CI: 2.0–4.9, *p* < 0.001). In conclusion, ARs are a quite common manifestation in COVID-19 patients, who are burdened by even worse prognosis. AR patients with AF presented the highest risk of mortality; thus, these patients may benefit from a more aggressive secondary preventive therapy and a closer follow up.

## 1. Introduction

Coronavirus disease (COVID-19) with severe acute respiratory syndrome (SARS) due to a novel Coronavirus (CoV-2) has rapidly spread worldwide [1]; as of 25 December 2021, over 278 million confirmed cases and nearly 5.3 million deaths were reported globally by the WHO [2]. The COVID-19 pandemic out broke in our regions at the end of February 2020, with a significant impact on cardiovascular hospitalization rate [3]. Arrhythmias (ARs) represent quite common complications of COVID-19 disease, particularly in patients admitted to intensive care units (ICU) [4]. Prevalence and type of ARs in COVID-19 patients vary across different studies [5]. Mechanisms of ARs development in COVID-19 patients are still not completely defined, although they have been already investigated in several papers [6,7,8]. Nevertheless, ARs onset in COVID-19 patients has been associated with increased mortality [9,10]. The aim of the present study was to investigate prevalence and impact on outcome of ARs at admission in hospitalized COVID-19 patients.

## 2. Materials and Methods

All-comer patients admitted to the Emergency Department of Modena University Hospital from 16 March to 31 December 2020 with confirmed COVID-19 pneumonia by positive nasopharyngeal swab and typical radiological features on chest X-ray were enrolled in the study. Clinical data, including outcome, were extracted from medical records. Venous and arterial blood samples for blood gas analysis were collected at the time of hospitalization and thereafter based on clinical indication. We used 12-lead ECG with 25 mm/s and 1 mV/cm calibration and 0.05–150 Hz filter setting. ECG traces were recorded and analysed offline. The following ECG parameters were considered: heart rate (HR), presence of ST-T abnormalities and corrected QT interval (msec), this latter was measured as the time between the start of the Q wave and the end of the T wave, corrected by HR according to Bazett’s formula. Rhythm and conduction alterations were also considered. The primary endpoint was 30-day mortality.

### Statistical Analysis

Continuous variables were expressed as medians and interquartile ranges, while categorical data were expressed as absolute values and proportions. Variables were compared between patient survivors and non-survivors at 30 days by using the Fisher exact test and the *t*-test, as appropriate. Survival curves were plotted using the Kaplan–Meier method with log-rank test and COX-regression model was applied. Analyses were performed with SPSS (Statistical Package for Social Science) software (v26, SPSS Inc., Chicago, IL, USA). For all the statistical analyses, *p* < 0.05 was considered significant.

## 3. Results

Baseline characteristics are summarized in Table 1.

**Figure 1:**
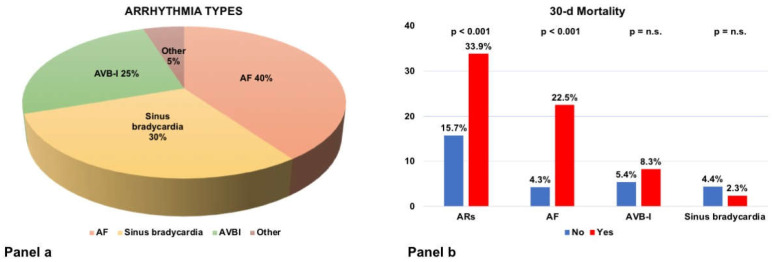
Panel (**a**): types of arrhythmias. Panel (**b**): 30-day mortality across ARs types.

Out of 902 COVID-19 patients enrolled, 637 (70.6%) underwent baseline 12-lead ECG at admission; of these, 122 (19.2%) were diagnosed with ARs. Compared to those without ARs, ARs patients were older (75.5 vs. 72, *p* < 0.001), with a more complex CV-history (hypertension, CA, CKD, AF (all *p* < 0.05). Symptoms and vital parameters did not differ between Ars and no-Ars patients. QTc interval prolongation (483 vs. 440 msec, *p* < 0.001) and conduction abnormalities (27.0% vs. 19.2%, *p* = 0.039) were more commonly found in Ars patients. These patients presented more frequently with lower levels of total white blood cells count (7.2 vs. 7.9, *p* = 0.042). AF (40.2%) and sinus bradycardia (29.5%) were the most common AR revealed at baseline 12-lead ECG (Figure 1, Panel a).

The primary endpoint occurred significantly higher in ARs patients (33.9% vs. 15.7%, *p* < 0.001, Figure 1, Panel b).

**Figure 2:**
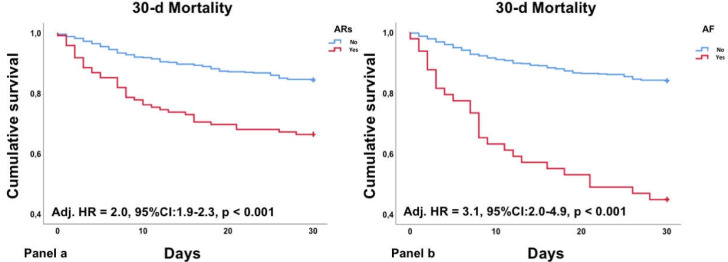
KM curves describing 30-day mortality for ARs (Panel (**a**)) and AF (Panel (**b**)).

AR patients presented an almost 3-fold risk of mortality compared to non-AR patients at 30d (Adj. OR = 2.8, 95%CI: 1.8–4.3, *p* < 0.001). After adjustment for significant baseline characteristics selected by a stepwise backward selection (Table 2), AR patients remained at increased risk of mortality (Adj. HR = 2.0, 95%CI: 1.9–2.3, *p* < 0.001, Figure 2a). Sub-group analysis revealed that among ARs patients, those with AF at admission presented the highest risk of 30-day mortality (Adj. HR = 3.1, 95%CI: 2.0–4.9, *p* < 0.001, Figure 2b).

## 4. Discussion

Cardiovascular diseases have been associated with worse prognosis in COVID-19 patients [11,12]. Recent evidence underlined whether arrhythmias may negatively affect outcome in this setting [13]. In this retrospective real-world cohort of COVID-19 patients, we confirmed a high short-term mortality, particularly in patients with baseline Ars, and we obtained the following novel key findings: (1) ARs at admission are quite common in COVID-19 infection and related to a higher risk of mortality; (2) among ARs, AF at admission has been associated with the highest risk of mortality; (3) myocardial biomarkers resulted higher in ARs patients compared to non-ARs patients, as an expression of a more severe disease with multi-organ-failure manifestation.

ARs at admission are frequently revealed in COVID-19 patients, particularly in critical conditions that require ICU admission [14]. ARs represent a common complication of COVID-19 disease with a prevalence as high as almost 50% in COVID-19 patients admitted to ICU [15]. Supraventricular ARs are commonly found in adult population and characterized up to one-fifth of COVID-19 patients [16], while ventricular ARs, such as ventricular tachycardia/fibrillation, are less frequently detected in this population. Bathla and colleagues reported an overall incidence of ARs of 7.5%, which is much higher in ICU patients [6]. The heightened risk of ARs in COVID-19 patients is related to different risk factors, primarily older age as well as comorbidities, the hyperinflammatory response typical of critical conditions, the direct effect of SARS-CoV-2 on myocardial tissue and the administration of pro-arrhythmic drugs. Being elderly is per se a risk factor for Ars development [17], and AF is the most common AR in adult population [18]. Race has been also associated with increased risk of ARs in COVID-19 patients in presence of a sodium channel variant that predispose to ARs [19]. Hypertension, diabetes, coronary artery disease and higher BMI have been also associated with higher risk of ARs development in COVID-19 patients [20,21]. Regardless the underlying mechanism, ARs have been associated with increased mortality in COVID-19 patients [12]. Baseline ECG abnormalities may be useful, in this setting, to predict the risk of cardiac arrest in COVID-19 patients [22]. In our cohort we found an incidence of baseline ARs around 20%, in line with data in the literature; these patients experienced a 2-fold risk of mortality, as reported in the results section. High heart rate, particularly sinus tachycardia, has been associated with higher inflammatory biomarkers and prolonged hospitalization in COVID-19 patients, but not with higher risk of thromboembolic events [23]. Sinus tachycardia resulted the most common ARs in a small cohort of COVID-19 patients, and survivors were less tachycardic than non-survivors [24]. In this context, heart rate variability (HRV) has been investigated and associated with the severity of COVID-19 disease, suggesting a potential role as non-invasive predictor for clinical outcome [25]. Recent evidence showed greater chances of survival in older COVID-19 patients with higher HRV [26].

AF is the most common AR in the adult population, with a global prevalence varying from 2% to 4% according to ESC guidelines [18]. Risk factors for AF include age, hypertension, COPD and other comorbidities. We demonstrated that COVID-19 patients with AF at baseline ECG presented almost a 3-fold risk of death compared to the non-AF-group. This finding is higher compared to previous data [27]. Reasons for this discrepancy may be related to the mean higher CHA2DSVASc of AF patients, which reflects the broad spectrum of comorbidities of this subpopulation. Although AF itself has been associated with being an independent predictor of mortality in COVID-19 patients, the distinction should be made between the role of AF and that of comorbidities on outcome. CHA2DS2VASc score, indeed, has been associated with increased mortality in COVID-19 patients, with an acceptable discriminatory power [28]. Nevertheless, AF seems to be a prognostic risk factor in COVID-19 patients independently from the burden of concomitant comorbidities [29]. Thus, AF may represent a useful clinical marker to identify COVID-19 patients at higher risk of death that should benefit from closer monitoring and more intensive therapy. Our study showed that AF at baseline ECG was characterized by a 3-fold higher risk of death compared to patients with no-AF at baseline ECG, whereas a history of AF had a 2-fold higher risk of death compared to patients without pre-existing AF. This suggests that AF at admission exerts a negative effect on COVID-19 patients’ outcome. The presence of AF in COVID-19 patients may precipitate and be perpetuated by hypoxia, with subsequent hemodynamic compromise and worse outcome, with the most prominent effect on the first 30 days.

Malignant ARs are strictly related to COVID-19 severity, particularly in critical patients with increased levels of cardiac biomarkers [30]. Older COVID-19 patients with tachyarrhythmias had a poorer prognosis compared to those without ARs [31]. Tachyarrhythmias, particularly sustained ventricular tachycardia, are more frequently revealed in COVID-19 patients presenting with acute coronary syndrome (ACS) [32]. TachyARs such as VT/VF are generally revealed in case of severe metabolic stress, while bradyARs could be a primary event contributing to mortality [33], but as reported in the results section, we encountered only few cases of this kind of ARs.

Cardiac biomarkers such as cTnI and NT-pro BNP have been investigated as potential prognostic tools in COVID-19 patients [34]. Patients with increased levels of cTnI reported a higher incidence of ARs, and recent evidence has suggested that the hyperinflammatory response induced by the SARS-CoV2 may precipitate ARs [8,35], and we reported higher levels of cTnI and BNP in ARs-patients.

### Limitations

Our results must be read considering some limitations. First, this is a retrospective single-centre cohort study, which limited the statistical power of our analyses. Moreover, given the logistical limitations at the onset of the pandemic infection, some laboratory data and ECG at admission were not collected in all patients. Echocardiography was not routinely performed in these patients. This study was designed considering COVID-19 patients at admission; thus, data regarding drugs’ administration or modality of data are lacking.

## 5. Conclusions

ARs are quite common at admission in COVID-19 infection, and have been associated with worse prognosis. In particular, AF at baseline ECG determined a significantly higher risk of mortality compared to other rhythms and even to the history of AF. Thus, baseline ARs should represent a simple clinical tool to stratify risk in COVID-19 patients and identify those subjects that may benefit from a closer follow-up and more aggressive therapies.

## Figures and Tables

**Table 1:** Baseline characteristics.

	Total (*n* = 902)	ARs (*n* = 122)	No-ARs (*n* = 515)	*p*
**Comorbidities**				
Age, years [IQR]	72.0 (24)	75.5 (22)	72.0 (29)	<0.001
Female sex, %	36.5	39.3	36.7	0.329
Hypertension, %	55.7	68.0	52.8	0.001
Diabetes, %	22.4	19.7	23.1	0.245
Obesity, %	12.7	11.5	13.0	0.388
COPD, %	9.4	10.7	9.1	0.355
Cancer, %	12.7	14.8	12.2	0.269
Previous AF, %	11.6	34.4	6.2	<0.001
CAD, %	13.7	23.8	13.6	0.005
PAD, %	8.0	11.5	9.9	0.354
CKD, %	10.6	18.0	9.5	0.008
Previous TE, %	3.2	6.6	3.1	0.069
Previous CVE, %	7.4	9.0	8.0	0.409
**Symptoms**				
Fever, %	81.6	79.5	82.1	0.289
Cough, %	44.9	38.5	46.6	0.065
Dyspnea, %	64.9	69.7	64.7	0.173
**Vital parameters**				
HR (bpm)	90	83 (29)	90 (23)	0.542
SO_2_ (%)	93	90 (9)	94 (5)	0.918
SBP (mmHg)	135	138 (64)	130 (40)	0.696
DBP (mmHg)	80	75 (33)	80 (20)	0.185
**ECG parameters**				
HR (bpm)	90 (22)	75 (46)	82 (22)	0.462
QTc (msec)	441 (39)	483 (99)	440 (30)	<0.001
aConduction	20.4	27.0	19.2	0.039
aRepolarization	5.6	7.4	5.2	0.236
**Biomarkers**				
cTnI (ng/L)	12 (30)	41 (56)	13 (57)	0.298
BNP (pg/mL)	89 (180.5)	287.5 (369)	92 (168)	0.160
WBC (10^3^/mm^3^)	6.6 (3.9)	7.2 (5.2)	7.9 (4.2)	0.042
CRP (mg/L)	5.7 (12.4)	14.1 (24.8)	13.1 (15.8)	0.986
LDH (U/L)	533 (227.3)	479 (473)	584 (183)	0.719
Uraemia (mg/dL)	42 (30)	48.5 (24)	42 (19)	<0.001

Legend: COPD = chronic obstructive pulmonary disease; AF = atrial fibrillation; CAD = coronary artery disease; PAD = peripheral artery disease; CKD = chronic kidney disease; TE = thromboembolism; CVE = cerebrovascular events; HR = heart rate; SO_2_ = oxygen saturation; SBP = systolic blood pressure; DBP = diastolic blood pressure; a = alterated; cTnI = cardiac troponin I; BNP = brain natriuretic peptide; WBC = white blood cell; CRP = C-reactive protein; LDH = lactate dehydrogenase.

**Table 2:** Baseline characteristics used for the multivariate analysis. ARs = arrhythmias; CAD = coronary artery disease.

	HR	95% CI	*p* Value
ARs	2.0	1.9–2.3	<0.001
Age > 65	6.1	3.1–11.9	<0.001
Hypertension	1.6	1.1–2.4	0.023
CAD	1.0	0.6–1.6	0.893
Comorbidities > 2	1.9	1.2–2.8	0.003

## Data Availability

Data files are available upon request.
